# Cross-country analysis of national mental health investment case studies in sub-Saharan Africa and Central, South and South-East Asia

**DOI:** 10.3389/frhs.2023.1214885

**Published:** 2023-07-18

**Authors:** Dan Chisholm, Yong Yi Lee, Phanindra Prasad Baral, Sadhana Bhagwat, Vladislav Dombrovskiy, Daniel Grafton, Anna Kontsevaya, Rumana Huque, Kenneth Kalani Okware, Alexey Kulikov, Kedar Marahatta, Patience Mavunganidze, Nasri Omar, Devi Prasai, Nadia Putoud, Elena Tsoyi, Jasmine Vergara

**Affiliations:** ^1^ Department of Mental Health and Substance Use, World Health Organization (WHO), Geneva, Switzerland; ^2^ Health Economics Division, School of Public Health and Preventive Medicine, Monash University, Melbourne, VIC, Australia; ^3^ School of Public Health, The University of Queensland, Brisbane, QLD, Australia; ^4^ Queensland Centre for Mental Health Research, Brisbane, QLD, Australia; ^5^ Epidemiology and Disease Control Division, Ministry of Health and Population, Kathmandu, Nepal; ^6^ WHO Country Office, Dhaka, Bangladesh; ^7^ Center for Healthcare Quality Assessment and Control, Moscow, Russia; ^8^ United Nations Development Programme, Istanbul, Turkey; ^9^ National Medical Research Center for Therapy and Preventive Medicine, Moscow, Russia; ^10^ Department of Economics, University of Dhaka, Dhaka, Bangladesh; ^11^ Mental Health Division, Ministry of Health, Kampala, Uganda; ^12^ United Nations Inter-Agency Task Force on the Prevention and Control of NCDs, Geneva, Switzerland; ^13^ WHO Country Office, Kathmandu, Nepal; ^14^ Mental Health Department, Ministry of Health and Child Care, Harare, Zimbabwe; ^15^ Division of Mental Health, Ministry of Health, Nairobi, Kenya; ^16^ Nepal Development Research Institute, Kathmandu, Nepal; ^17^ Division of Country Health Programmes, WHO Regional Office for Europe, Copenhagen, Denmark; ^18^ WHO Country Office, Manila, Philippines

**Keywords:** mental health, health financing, return on investment, benefit-cost, global health

## Abstract

**Introduction:**

Despite the increasing interest in and political commitment to mental health service development in many regions of the world, there remains a very low level of financial commitment and corresponding investment. Assessment of the projected costs and benefits of scaling up the delivery of effective mental health interventions can help to promote, inform and guide greater investment in public mental health.

**Methods:**

A series of national mental health investment case studies were carried out (in Bangladesh, Kenya, Nepal, Philippines, Uganda, Uzbekistan and Zimbabwe), using standardized guidance developed by WHO and UNDP and implemented by a multi-disciplinary team. Intervention costs and the monetized value of improved health and production were computed in national currency units and, for comparison, US dollars. Benefit-cost ratios were derived.

**Findings:**

Across seven countries, the economic burden of mental health conditions was estimated at between 0.5%–1.0% of Gross Domestic Product. Delivery of an evidence-based package of mental health interventions was estimated to cost US$ 0.40–2.40 per capita per year, depending on the country and its scale-up period. For most conditions and country contexts there was a return of >1 for each dollar or unit of local currency invested (range: 0.0–10.6 to 1) when productivity gains alone are included, and >2 (range: 0.4–30.3 to 1) when the intrinsic economic value of health is also considered. There was considerable variation in benefit-cost ratios between intervention areas, with population-based preventive measures and treatment of common mental, neurological and conditions showing the most attractive returns when all assessed benefits are taken into account.

**Discussion and Conclusion:**

Performing a mental health investment case can provide national-level decision makers with new and contextualized information on the outlays and returns that can be expected from renewed local efforts to enhance access to quality mental health services. Economic evidence from seven low- and middle-income countries indicates that the economic burden of mental health conditions is high, the investment costs are low and the potential returns are substantial.

## 1. Introduction

Mental health is a neglected but major challenge for public health and sustainable development. Whether viewed from the perspective of disease burden, social inequality or human rights, there is an evident and increasing need to revitalize efforts to strengthen the whole system of mental health promotion, protection and care (1). Additionally, the economic consequences of diminished or foregone mental health are substantial. In a study conducted for the World Economic Forum (2), the projected global economic losses attributable to mental, neurological and substance use conditions between 2011 and 2030 were estimated to be US$ 16 trillion, and in a subsequent study led by WHO, it was estimated that common mental disorders alone cost the global economy US$ 1 trillion per year (3).

These studies were undertaken before the COVID-19 pandemic, which greatly increased population exposure to adversity and other risk factors for mental health, and led to a rapid rise in the prevalence of common mental health conditions such as depression and anxiety (4). The sheer extent of mental health impacts brought about by the pandemic—as well as other acute or emerging crises and emergencies (including conflict, migration, and climate change)—has led to a new level of political attention and commitment to mental health. Yet financial commitment still lags far behind, with often very meagre levels of investment in mental health services and systems, especially in lower-income countries with heavily constrained health and welfare budgets (5).

The benefits of investing in mental health extend well beyond better care and services for people with mental health conditions, and include: greater public awareness, understanding and literacy about the causes and effects of better or worse mental health, which can directly help to reduce stigma and discrimination against people with mental health conditions; enhanced opportunities for nurturing and protecting cognitive, emotional and social capacities as well as educational outcomes of children and adolescents; and reduced high cost to businesses and national economies due to lost productivity (6).

Assessing the economic case for increased mental health care has largely been focused on estimating the costs and cost-effectiveness of a range of treatment strategies for common or priority mental health conditions [see for example (7–9)]. This has helped generate an evidence base for investment decisions at the national level. However, such studies have typically focused on specific clinical interventions and, unlike cost-benefit or return on investment (ROI) analysis, do not capture the wider social or economic benefits that accompany improvements to health and functioning. To respond to these limitations, WHO and UNDP developed guidance for and supported the application of national mental health investment case (MHIC) studies.

This paper provides a cross-country analysis and overview of seven completed MHICs carried out across Africa (Kenya, Uganda, Zimbabwe) and Asia (Bangladesh, Nepal, Philippines, Uzbekistan) (8, 10–12)^1^^–^^3^. Table 1 provides a summary of key socioeconomic characteristics of each country.

**Table 1:** Socioeconomic characteristics of countries.

	Bangladesh	Uganda	Uzbekistan	Kenya	Nepal	Philippines	Zimbabwe
Population (in millions)	164.7	47.03	34.2	47.5	30.3	109.7	15.4
World Bank income group	Lower-middle income	Low income	Lower-middle income	Lower-middle income	Lower-middle income	Lower-middle income	Lower-middle income
GDP per capita [US$]	1,969	731	1,478^a^	2,080	1,189	3,016^a^	1,667
GDP per employed person [US$]	5,044	2,017	3,642^a^	5,598	2,454	7,527^a^	7,695
Lost output per worker over 10 years [US$]	57,175	19,408	38,783^a^	55,892	25,852	84,356^a^	85,424
Total health expenditure (US$,)	46	32	99	83	53	142	103
Labor force participation rate (15 + years) [%]	56%	66%	62%	69%	77%	62%	46%

All data are for the year 2019–2020, unless otherwise specified.

^a^ 2018 data.

## 2. Methods

### 2.1. Analytical framework

All of the national studies reported here used and followed the methodological guidance note developed and issued by WHO and UNDP (6) to provide a consistent and structured approach for making national cases for investment in mental health, as described further below. Investment cases are part of a WHO-UNDP Joint Programme to catalyze multisectoral action in Member States to reduce the burden of noncommunicable diseases (NCDs) and mental health conditions. The Joint Programme is part of the larger work of a UN Inter-Agency Task Force on the Prevention and Control of NCDs. The guidance note provides an overview of how to make an investment case, covering the principles and practice of analyzing return on investment (ROI) and its application to the mental health sector. The guidance note further includes sections on intervention costing, estimation of population-level health impacts, monetization of benefits and ROI metrics. ROI analysis offers a convenient, comparable measure of the efficiency of one or more investment choices, expressed as the expected flow of costs and (monetized) benefits resulting from an investment of resources. Expression of both the costs and the benefits of an innovation or intervention in the same units (money) makes investment decisions straightforward, indicating that, if the benefits of an investment are larger than the costs, it is sound.

The guidance note also offers guidance for undertaking an institutional context analysis (ICA), which enables institutions and/or countries to appraise the political context around implementing priority interventions from the investment case. It reveals where consensus, political appetite, opportunities, challenges and barriers lie.

Each country established a team of local experts to collect and review data, agree on which conditions and interventions to focus on; and to discuss analytical choices, assumptions and preliminary results. These national teams were supported by an international team made up of health economists, mental health specialists and secretariat staff of UNDP, WHO and the Inter-Agency Task Force.

Mental health conditions included in the investment cases ranged from severe mental disorders (psychosis and bipolar disorder), common mental and neurological disorders (depression, anxiety and epilepsy) and alcohol use disorders. Evidence-based treatments for these conditions were identified on the basis of WHO guidelines and associated cost-effectiveness analyses (13, 14). In addition, analysis was carried out for bans of highly hazardous pesticides for preventing suicide (15) and school-based social and emotional learning (SEL) for reducing the risk of depression, anxiety and suicide (16).

The time horizon selected by the country teams for the analysis ranged from 10 years (in Bangladesh, Uganda and Uzbekistan) to 20 years (in the other countries), based on the longer-term planning cycle within which the investment case work was expected to contribute. Country results were accordingly grouped by time horizon, since this has a direct influence on average annual costs and benefits (i.e., lower annual values for the longer scale-up period). Intervention costs and benefits were considered for this period only, and not beyond the final year of scale-up.

### 2.2. Epidemiology, intervention effects and service coverage for mental health conditions

**Figure 1:**
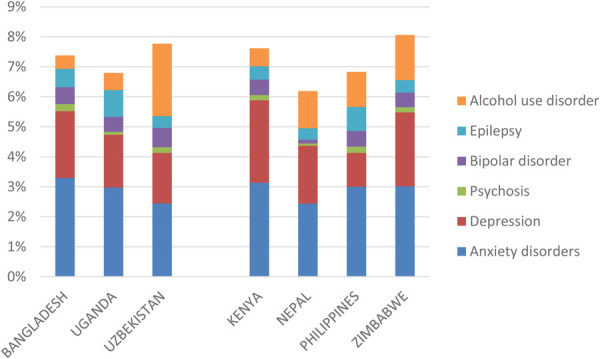
Prevalence of assessed mental, neurological and substance use (MNS) conditions [%].

Age- and sex-specific incidence, prevalence, remission and mortality rates—as well as levels of disability or functioning—for each included condition were based on local survey data (if available, such as in Bangladesh and Nepal) or from country-specific estimates obtained from the Global Burden of Disease study (GBD Results tool; https://ghdx.healthdata.org/gbd-2019). Figure 1 shows the prevalence of assessed conditions in each of the countries (as a percentage of all age groups in the population); the cumulative prevalence accounts for some but not all comorbidities that exist between these conditions and some but not all mental, neurological and substance use conditions in the population (for example, the national mental health survey in Bangladesh also includes estimated prevalence of conduct disorder, obsessive-compulsive disorder, personality disorder and somatoform disorders, among others), so should be interpreted accordingly.

Intervention effect sizes were based on estimates available from WHO's cost-effectiveness work programme, typically expressed as a percentage improvement in the rate of incidence, remission, mortality or functioning (7). These effect sizes were subsequently attributed to the proportion of people with the mental health condition capable of benefiting from the specific intervention (such as psychological treatment for moderate-severe depression), and then applied to current, increasing and target levels of expected coverage in the population. Composite baseline and target coverage rates for the different mental health conditions are shown in Table 2. These were based on survey data (if available) or agreed upon through a process of local expert consultation and consensus.

**Table 2:** Intervention coverage (baseline | target), by MNS condition [%].

	10 year scale-up period			20 year scale-up period			
	Bangladesh baseline | target	Uganda baseline | target	Uzbekistan baseline | target	Kenya baseline | target	Nepal baseline | target	Philippines baseline | target	Zimbabwe baseline | target
Anxiety disorders	15% | 58%	10% | 48%	4% | 29%	8% | 48%	10% | 48%	8% | 48%	11% | 48%
Depression	11% | 50%	11% | 50%	3% | 32%	8% | 50%	8% | 50%	8% | 50%	9% | 50%
Psychosis	30% | 70%	30% | 70%	65% | 90%	28% | 90%	24% | 90%	28% | 90%	28% | 90%
Bipolar disorder	29% | 60%	30% | 70%	10% | 60%	28% | 90%	23% | 70%	28% | 90%	27% | 70%
Epilepsy	42% | 60%	42% | 60%	7% | 70%	33% | 90%	33% | 90%	43% | 90%	43% | 90%
Alcohol use disorder	26% | 100%	26% | 100%	13% | 50%	25% | 100%	27% | 100%	21% | 100%	84% | 100%

To estimate the population-level health impact of these interventions, a strategic costing and planning tool developed by WHO and other UN agencies called the OneHealth tool was used (https://avenirhealth.org/software-onehealth.php), which enabled calculation of the number of healthy years of life lived in the population at current and target levels of coverage. Healthy life years include both expected changes in life expectancy (e.g., as a result of a decrease in the case fatality rate after introduction of a pesticide ban), and also non-fatal health outcomes (e.g., reduced incidence or duration of depressive episodes after treatment).

### 2.3. Economic burden and intervention costs

Although assessment of the economic burden or consequences of mental health conditions is not a pre-requisite for ROI analysis, it provides relevant policy information and context. Economic costs were therefore established, both in terms of direct mental health expenditure and in terms of productivity losses due to absenteeism, presenteeism and premature mortality. Mental health expenditure was based on available national health accounts data, and as reported through periodic international surveys such as the WHO mental health Atlas (5). For productivity losses, which includes estimates of both absenteeism and presenteeism, data were taken from the World Health Surveys on the average number of complete and partial days out of role per year for each of the selected conditions, respectively (17), and subsequently applied to the number of expected cases in the currently employed adult workforce [see methodological guidance note developed and issued by WHO and UNDP (6) for more details]. Similarly, annual deaths attributable to each condition were multiplied by the average GDP per employed worker to generate an estimate of productivity loss due to premature mortality.

For intervention costing, the main categories of resource cost were: inpatient care; outpatient and primary care (including psychosocial support and psychological treatment); medication; programme costs and shared health system resources, including programme management, administration and monitoring at the national and sub-national level, training and supervision of non-specialist workers, and mental health promotion and communications. Unit costs for each resource item were obtained from locally available data sources to the extent possible (e.g., medication prices, worker salaries, etc.) and supplemented by country-specific estimates from the WHO-CHOICE database (18).

### 2.4. Economic benefits and return on investment analysis

Both the intrinsic value of improved mental health and well-being, as well as its instrumental value (e.g., being able to form and maintain relationships, study, work or pursue leisure interests and to make decisions in everyday life) were estimated. Productivity gains resulting from interventions for treating depression, anxiety and alcohol use disorders include increased labour force participation (by avoided mortality and illness) and reduced absenteeism and presenteeism (3). As data on labour force outcomes for people with psychosis, bipolar disorder and epilepsy is limited, a more indirect method was used that relied on taking the total healthy life years gained by an intervention and multiplying this by the Gross Domestic Product (GDP) per capita in each country, as recommended in a *Lancet* Commission on investing in health (19). Productivity gains due to reduced absenteeism and presenteeism were not estimated for the school interventions, as they are not relevant to people of non-working age.

In addition to calculating the productivity gains attributable to each mental health intervention, separate estimates were calculated for the intrinsic value of improving health as an end in itself; these gains are reported here as the social (as opposed to productivity) effects of intervention. Latest recommendations for investing in health using a ‘full-income’ approach and for carrying out cost-benefit analysis in the health sector propose that the social value of one healthy life year gained be valued at 1.5 times GDP per capita (19, 20); accordingly, each healthy life year gained through intervention was multiplied by this monetary value.

The return for each intervention was calculated by comparing the instrumental and intrinsic economic benefits produced by the intervention with the total costs of setting up, implementing and scaling-up the interventions over time. Projected costs and economic benefits were estimated using the net present value approach and a 3% annual discount rate (6). The summary metric used in the analysis presented here is the benefit-to-cost ratio, defined as the present value of total health and/or productivity gains divided by the present value of total intervention costs.

## 3. Results

### 3.1. Economic burden of mental health conditions

**Figure 2:**
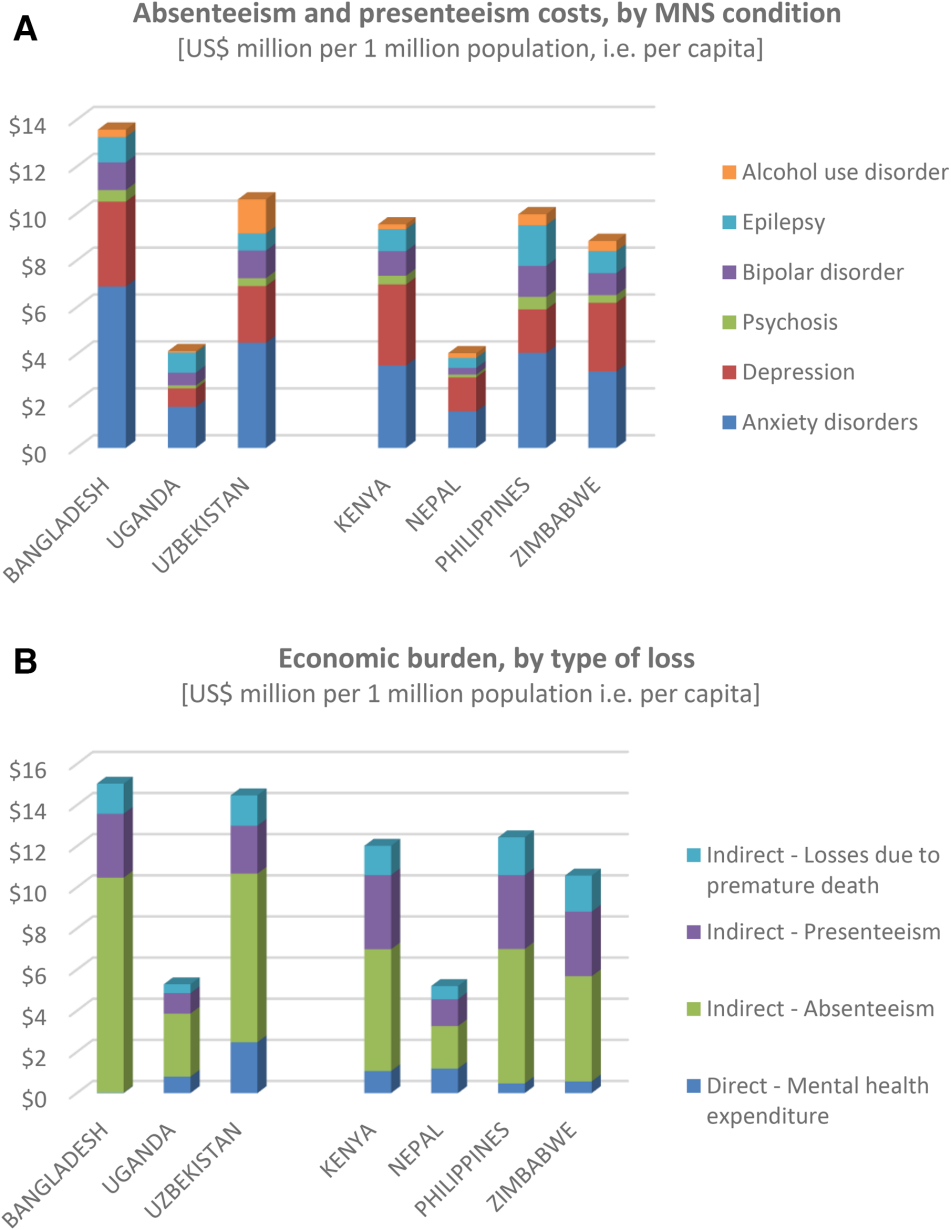
Economic burden of mental health conditions.

The economic losses associated with mental health conditions are considerable. As shown in Figure 2A, the economic consequences of lost productivity—made up of the economic value of fully lost or partially lost work days—amount to as much as US$10 or more per head of population annually [inter-country range: US$ 4.00–13.50]; depression and anxiety disorders represent the main contributors to this productivity loss on account of their relatively high prevalence in the population.

When these costs are combined with losses due to premature death and with the expenditures on mental health care, the total annual attributable economic burden is estimated at close to US$ 5 per capita in the countries with the lowest GDP per capita (Nepal and Uganda) and US$ 10–15 per capita in the remaining five countries (Figure 2B). Across the seven countries, these amounts are equivalent to 0.4%–1.0% of GDP. Productivity losses account for the large proportion of total economic burden.

### 3.2. Costs and health effects of scaled-up intervention packages

**Figure 3:**
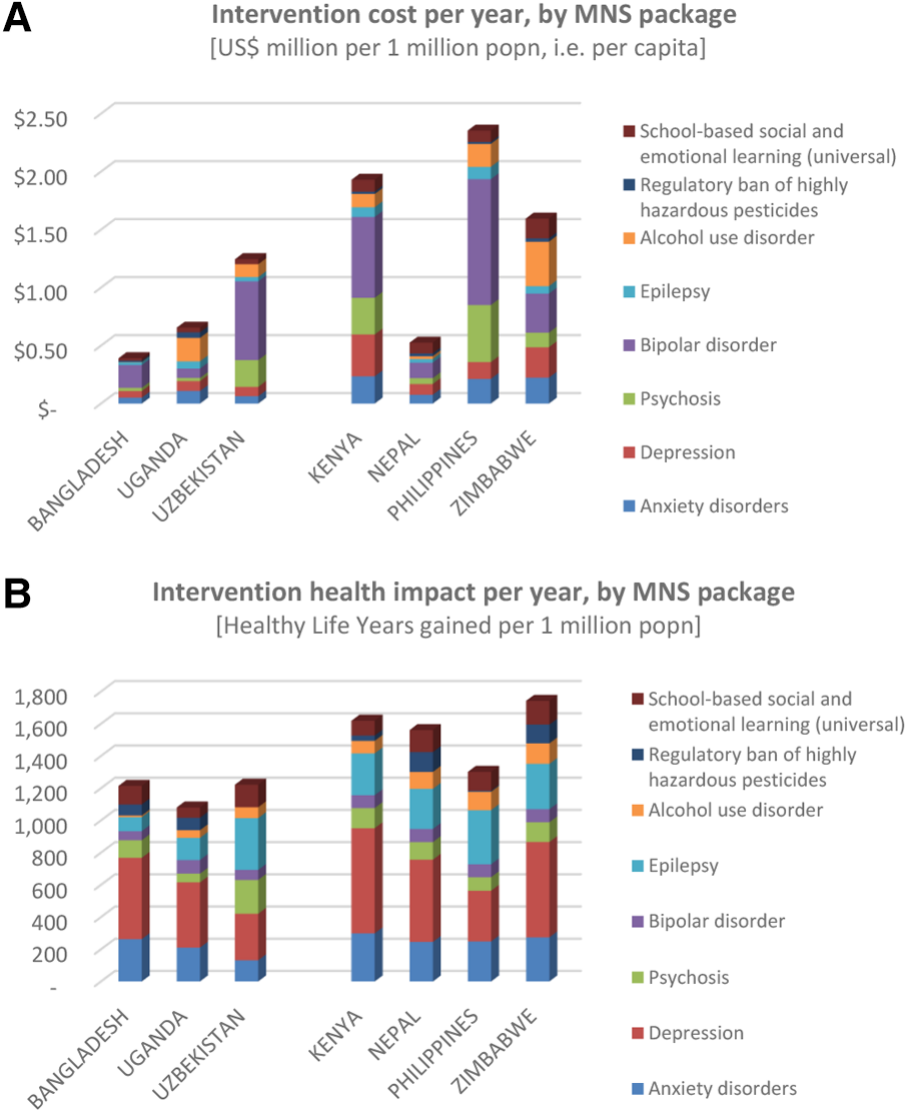
Costs and health effects of scaled-up intervention packages.

Compared to the economic losses associated with mental health conditions, the estimated costs of massively scaled-up delivery of evidence-based mental health intervention packages are very low, ranging from below US$ 0.50 per capita in Bangladesh and Nepal, up to US$1.50–2.50 in Kenya, Philippines and Zimbabwe (Figure 3A). Population-based prevention strategies (i.e., SEL programmes in schools and pesticide bans) carried the lowest implementation costs, while interventions for bipolar disorder had the highest costs (mainly due to relatively high costs of mood-stabilizing medication and the adoption of an intensive level of outpatient treatment and follow-up support for this condition in many of the countries).

The expected health gains generated by the scaled-up delivery of these intervention packages are shown in Figure 3B. The number of healthy life years gained per year per one million population was found to range between 1,100–1,250 in countries adopting a 10-year time horizon (and relatively lower target coverage levels), and 1,300–1,750 in those with a 20-year horizon (and relatively higher target coverage levels).

### 3.3. Economic benefits and return on investment

**Figure 4:**
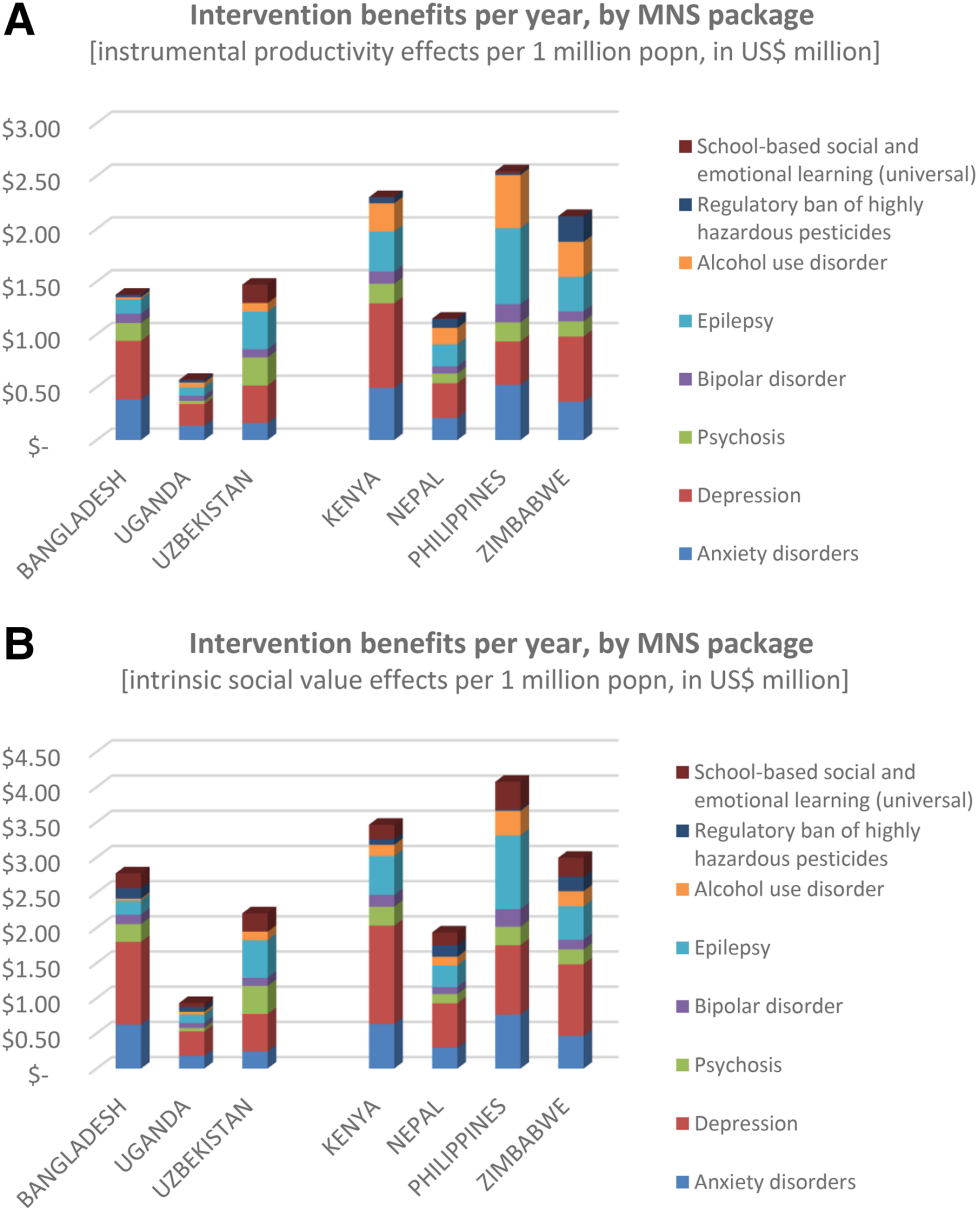
Economic benefits of scaled-up intervention packages.

In terms of restored productivity alone, scaled-up delivery of the mental health intervention packages led to an estimated US$ 1.5–2.5 million per one million population (i.e., US$ 1.5–2.5 per capita or head of population), mainly arising from the common mental, neurological and substance use conditions involving anxiety, depression, alcohol use disorders and epilepsy (Figure 4A). The exception was Uganda (US$ 0.57 per head of population), owing to the low GDP per capita there. Similarly for the social benefits accorded to the intrinsic value of better health, intervention scale-up led to substantially lower gains in Uganda (US$ 0.94) than elsewhere (US$ 2–4) (Figure 4B).

Relating these benefits back to the costs incurred in securing them produces a range of benefit-cost ratios that are presented in Table 3. Across the seven countries, and in terms of productivity gains alone, each dollar invested in the intervention packages produces a benefit of more than US$ 1 in a majority of countries and for most of the conditions assessed, thereby providing an economic rationale for investment. Benefit-cost ratios ranged from 1.2–7.2 to 1 for anxiety and epilepsy, 2.2–9.7 to 1 for depression, and 0.6–6.2 to 1 for psychosis. Ratios were lowest for bipolar disorders (0.1–0.6) and in Uganda, which is a reflection of the relatively low GDP per worker used to monetise rates of workforce productivity. By contrast, benefit-cost ratios for Bangladesh were relatively high, owing to very low costs of intervention scale-up but a much higher GDP per worker than Uganda.

**Table 3:** Benefit-cost ratios of mental health intervention packages.

	BAN	UGANDA	UZB	KENYA	NEPAL	PHI	ZIM
Benefit-cost ratio [productivity effects alone]							
Anxiety disorders	7.2	1.2	2.5	2.1	2.7	2.5	1.6
Depression	9.7	2.4	4.4	2.2	3.5	2.8	2.4
Psychosis	6.2	1.1	1.2	0.6	1.8	0.4	1.1
Bipolar disorder	0.4	0.6	0.1	0.2	0.5	0.2	0.3
Epilepsy	5.8	1.3	9.7	4.5	6.6	6.7	5.1
Alcohol use disorder	10.6	0.2	0.7	2.3	6.6	2.5	0.9
Regulatory ban of highly hazardous pesticides	2.0	0.5		2.9	3.6	1.1	8.3
School-based social and emotional learning (universal)	0.1	0.0	4.0	0.0	0.0	0.2	0.0
Benefit-cost ratio [productivity + social effects]							
Anxiety disorders	19.1	2.9	6.4	4.8	6.7	6.1	3.7
Depression	30.3	6.5	11.0	6.1	10.3	9.6	6.2
Psychosis	15.4	2.9	2.9	1.4	4.4	0.9	2.8
Bipolar disorder	1.1	1.5	0.3	0.4	1.2	0.4	0.7
Epilepsy	14.5	3.2	24.3	11.1	16.2	16.6	12.6
Alcohol use disorder	24.0	0.4	1.8	3.7	12.0	4.3	1.4
Regulatory ban of highly hazardous pesticides	14.5	1.9		6.6	10.2	2.4	15.4
School-based social and emotional learning (universal)	13.8	1.4	9.9	2.1	2.0	4.2	1.6

All figures presented are the estimated ratio of benefit to one unit of cost (e.g. for US$ 1 invested in anxiety disorders in Bangladesh, there is a benefit of US$ 7.2 from productivity gains).

Addition of the social value associated with restored health significantly increases benefit-cost ratios; for example they now range from 6.2–30.3 to 1 for depression and 3.3–24.3 to 1 for epilepsy. Ratios remain low for bipolar disorder (0.3–1.5) and in Uganda (0.4–6.5).

Regarding population-based strategies, regulatory bans of highly hazardous pesticides produced benefit-cost ratios of 0.5–8.3 to 1 (productivity effects alone) and 1.9–15.4 to 1 (productivity and social effects). The productivity effects of scaled-up delivery of school-based SEL programmes were minimal since the target group are not of working age, but the health and social impacts were estimated to result in benefit-cost ratios of 1.4–13.8 to 1 across the seven countries.

## 4. Discussion

### 4.1. Key findings

In the context of increasing political but static financial commitment to mental health at the global level, assessment of the economic consequences and options for scaled-up investment at the national level can help to ‘make the case’ and stimulate new resource generation and partnerships. An earlier global ROI study for scaled-up treatment of depression and anxiety (3) garnered significant interest and has been used to advocate for greater investment at the international level. More recently, a comprehensive and comparative ROI assessment has been completed for adolescent mental health, which indicated that implementing a selected set of interventions offers a return on investment of more than 20 to 1 (21).

The completion of seven country-level MHICs in a range of socioeconomic and cultural contexts across Central, Southeast and South Asia and also sub-Saharan Africa represents a significant addition to the existing evidence base. It also provides an opportunity to reflect on and compare the process, application and results of these studies. Across the seven countries, implementing and completing these studies was itself a valuable vehicle for improved multi-sectoral dialogue, collaboration and planning, a process that was enriched not only by the establishment of local working groups but also through the conduct of several interviews held with local stakeholders to understand the current policy and practice context.

The output of these studies are unique to each country's context, but there are a number of common threads running through the results. First, in all countries the economic burden of mental health conditions is substantial, typically amounting to 0.5%–1.0% of GDP. Second, and by contrast, the level of investment needed to scale-up mental health intervention packages was found to be very modest, ranging from US$ 0.50–2.50 per year per head of population, equivalent to 0.9%–2.5% of total health expenditure and 0.03%–0.14% of GDP. Thirdly, the monetised benefits of this intervention scale-up were generally found to exceed investment costs, often by a great margin (such as for anxiety, depression and epilepsy treatment). However, there were exceptions, including for certain conditions (bipolar disorder) and particular countries (Uganda). The observed variation between countries is attributable to multiple factors, including the prevailing prices and unit costs of health care production, existing levels of intervention coverage and, especially, GDP (per capita and per worker).

### 4.2. Limitations and lessons

The results of these studies need to be interpreted in light of several limitations and shortcomings, starting with the paucity of local data on disease prevalence and mortality, the current effective coverage of interventions, and productivity losses in the workplace. In a number of countries, there are no nationally-representative epidemiological survey data on mental health at all. As a result, many parameters used in the economic analysis were drawn from international data sources (such as the Global Burden of Disease study or international research studies on intervention effectiveness) and then subjected to consultation with and review by local experts.

A vital aspect of ROI analysis relates to productivity losses and potential gains from intervention, plus their valuation in monetary terms. Not all possible productivity losses were considered (e.g., unpaid household production losses and care-giver time), and average GDP per worker was used as a proxy (but not necessarily accurate) measure of workforce productivity, especially in populations with high levels of informal employment. In addition, and as indicated, future productivity gains associated with school-based programmes for adolescents yet to reach working age were not estimated. Recent research incorporated into an ROI analysis for adolescent mental health interventions enabled such long-term benefits to be captured, which is an important methodological development (21).

Countries were also effectively constrained in the choice of conditions and interventions to include, since not all evidence-based strategies for prevention and management of mental, neurological and substance use conditions have been incorporated into the OneHealth tool used to generate estimates of population-level impact. For example, it was not possible to include estimation of potential returns to investment for treatment of drug use disorders, despite the interest of some countries in doing so. Similarly, only two population-based measures were assessed (a ban on highly hazardous pesticides, and social and emotional learning programmes in schools).

### 4.3. Policy implications

The generation and publication of these national MHICs complements existing economic evidence at the regional level on the costs and cost-effectiveness of scaled-up mental health intervention packages (14). A particular feature and advantage of these MHICs is that they capture and quantify benefits beyond the health system alone, in particular the restored workforce productivity arising from improved mental health. As such, the results from these studies offer national decision-makers in and beyond the health sector with fresh data and information that can be used in multi-sectoral planning, prioritisation and resource allocation processes, including but not limited to specification of essential packages of health care as part of universal health coverage (UHC).

The information presented in the MHICs also provides further impetus and justification for these countries to enact legislation, develop policies, increase budgets and reform health services, so that they enable and support scaled-up implementation of the evidence-based intervention packages assessed. The Institutional Context Analysis carried out and reported alongside the economic analysis generated new interest, understanding and engagement among national stakeholders, and ensured that each investment case was embedded in local realities and sectoral plans. Recommended actions outlined in the final reports for each country included: shifting attention and resources away from institutions to more community-based approaches to mental health care provision; integrating mental health into primary health care as well as prioritised programmes such as HIV, TB, and NCDs; and consideration of innovative funding mechanisms or pathways for more sustainable and equitable mental health financing.

## Data Availability

The raw data supporting the conclusions of this article will be made available by the authors, without undue reservation.

## Footnotes

^1^WHO. Prevention and management of mental health conditions in Bangladesh: The case for investment. Final Report (forthcoming publication). (2023).

^3^WHO. Prevention and management of mental health conditions in Uganda: The case for investment. Final Report (forthcoming publication). (2023).

## References

[1] WHO. World mental health report; transforming mental health for all. Geneva: WHO (2022). Available at: https://www.who.int/publications/i/item/9789240049338 (Accessed April 28, 2023).

[2] Bloom DE Cafiero ET Jané-Llopis E Abrahams-Gessel S Bloom LR Fathima S The global economic burden of noncommunicable diseases. Geneva: World Economic Forum (2011). Available at: https://www3.weforum.org/docs/WEF_Harvard_HE_GlobalEconomicBurdenNonCommunicableDiseases_2011.pdf (Accessed July 14, 2023).

[3] Chisholm D Sweeny K Sheehan P Rasmussen B Smit F Cuijpers P Scaling up treatment of depression and anxiety: a global return on investment analysis. Lancet Psychiatry. (2016) 3:415–24. 10.1016/S2215-0366(16)30024-4 27083119

[4] COVID-19 Mental Disorders Collaborators. Global prevalence and burden of depressive and anxiety disorders in 204 countries and territories in 2020 due to the COVID-19 pandemic. Lancet. (2022) 398:1700–12. 10.1016/S0140-6736(21)02143-7 PMC8500697 34634250

[5] WHO. Mental health atlas 2020. Geneva: WHO (2021). Available at: https://www.who.int/publications/i/item/9789240036703 (Accessed April 28, 2023).

[6] WHO and UNDP. Mental health investment case: A guidance note. Geneva: WHO (2021). Available at: https://www.who.int/publications/i/item/9789240019386 (Accessed April 28, 2023).

[7] Chisholm D Saxena S. Cost effectiveness of strategies to combat neuropsychiatric conditions in sub-Saharan Africa and South East Asia: mathematical modelling study. Br Med J. (2012) 344:e609. 10.1136/bmj.e609 22389339 PMC3292519

[8] WHO. Prevention and management of mental health conditions in the Philippines: The case for investment. Geneva: WHO (2021). Available at: https://www.undp.org/philippines/publications/prevention-and-management-mental-health-conditions-philippines-case-investment (Accessed July 14, 2023)

[9] Knapp MRJ Hong G (2020). Economics and mental health: the current scenario. World Psychiatry, 19: 3–14. 10.1002/wps.20692 31922693 PMC6953559

[10] Republic of Kenya Ministry of Health. Providing evidence for the long-term health, social and economic benefits of investment in mental health in Kenya. Nairobi, Kenya: Ministry of Health (2021). Available at: https://mental.health.go.ke/download/kenya-mental-health-investment-case-2021/ (Accessed April 28, 2023).

[11] WHO. Prevention and management of mental health conditions in Uzbekistan: The case for investment. Geneva: WHO (2021). Available at: https://apps.who.int/iris/handle/10665/348760 (Accessed April 28, 2023).

[12] WHO. Prevention and management of mental health conditions in Zimbabwe: The case for investment. Final Report. (2022). Available at: https://www.who.int/publications/m/item/investment-case-for-zimbabwe-(1) (Accessed April 28, 2023).

[13] WHO. mhGAP intervention guide mental health gap action programme version 2.0. Geneva: WHO (2016). Available at: https://www.who.int/publications/i/item/9789241549790 (Accessed July 14, 2023). 27786430

[14] WHO. Menu of cost-effective interventions for mental health. Geneva: World Health Organization (2021). Available at: https://www.who.int/publications/i/item/9789240031081 (Accessed April 28, 2023).

[15] Lee YY Chisholm D Eddleston M Gunnell G Fleischmann A Konradsen F The cost-effectiveness of banning highly hazardous pesticides to prevent suicides due to pesticide self-ingestion across 14 countries: an economic modelling study. Lancet Global Health. (2021) 9:e291–e300. 10.1016/S2214-109X(20)30493-9 33341152 PMC7886657

[16] Lee YY Skeen S Melendez-Torres GJ Laurenzi CA Van Ommeren M Fleischmann A School-based socio-emotional learning programs to prevent depression, anxiety and suicide among adolescents: a global cost-effectiveness analysis. Epidemiol Psychiatr Serv. (2023) 32:E46. 10.1017/S204579602300029X PMC10477081 37434513

[17] Alonso J Petukhova M Vilagut G Chatterji S Heeringa S Üstün TB Days out of role due to common physical and mental conditions: results from the WHO world mental health surveys. Mol Psychiatry. (2011) 16:1234–46. 10.1038/mp.2010.101 20938433 PMC3223313

[18] WHO. WHO-CHOICE estimates of cost for inpatient and outpatient health service delivery. (2021). Available at: https://www.who.int/publications/m/item/who-choice-estimates-of-cost-for-inpatient-and-outpatient-health-service-delivery (Accessed April 28, 2023).

[19] Jamison DT Summers LH Alleyne G Arrow KJ Berkley S Binagwaho A Global health 2035: a world converging within a generation. Lancet. (2013) 382:1898–955. 10.1016/S0140-6736(13)62105-4 24309475

[20] Robinson L Hammitt J Cecchini M Chalkidou K Claxton K Cropper M Reference case guidelines for benefit-cost analysis in global health and development. Boston, MA: Harvard T.H. Chan School of Public Health (2019). Available at: https://sites.sph.harvard.edu/bcaguidelines/ (Accessed April 28, 2023).

[21] Stelmach R Kocher EL Kataria I Jackson-Morris AM Saxena S Nugent R. The global return on investment from preventing and treating adolescent mental disorders and suicide: a modelling study. BMJ Glob Health. (2022) 7(6):e007759. 10.1136/bmjgh-2021-007759 35705224 PMC9240828

